# Poisoning from Subnitrate of Bismuth

**Published:** 1895-01-05

**Authors:** 


### TOXICOLOGICAL NOTE.

[We shall be glad to receive, at our Office, 428, Strand, London, W.C., from the manufacturers, specimens of all new preparations and drugs which may be brought out from time to time.]

### POISONING FROM SUBNITRATE OF BISMUTH.

Dr. Wilson[1] reports a case in which poisonous symptoms supervened from absorption of subnitrate of bismuth. A girl, fifteen years old, suffering from a burn on the back, having an area of about sixteen inches by fifteen inches, had a dressing of bismuth subnitrate applied to it every second day. At the end of eight days nausea, headache, vomiting, high temperature, quick pulse, and a urinous odour of the breath appeared. There was slight albuminuria, and a black line along the margin of both gums, with diarrhœa and œdema of the legs. Death occurred eight days later with continuance of the same symptoms. The bismuth was tested for lead and arsenic, with negative results. Such cases are extremely rare, as bismuth is seldom used as a dressing for large raw surfaces, but a few years ago, when trial was being made of it on a pretty extensive scale on the Continent as a surgical dressing, several similar results were reported and led to the abandonment of its use. Experiments on animals, in which a soluble double salt of bismuth was given by a vein or hypodermically, have made us acquainted with exactly similar effects following its administration, and which are undoubtedly due to the action of the bismuth. Such effects are not seen when it is given by the mouth, as only a minute quantity is absorbed from the intestinal canal, quite insufficient to cause poisoning. Irritative and poisonous symptoms were sometimes seen formerly after giving bismuth salts, but these were long since shown to be due to contamination with l[illegible] or arsenic, and have disappeared with improveme[illegible] manufacture.

[1] New York Med. Journal, Jan. 20, 1894.

